# Case report: *Pneumocystis jirovecii* pneumonia in a severe case of Aicardi–Goutières syndrome with an *IFIH1* gain-of-function mutation mimicking combined immunodeficiency

**DOI:** 10.3389/fimmu.2022.1033513

**Published:** 2023-01-04

**Authors:** Mojca Železnik, Aneta Soltirovska Šalamon, Maruša Debeljak, Aleš Goropevšek, Nataša Šuštar, Damjana Ključevšek, Alojz Ihan, Tadej Avčin

**Affiliations:** ^1^ Department of Neonatology, Children’s Hospital, University Medical Centre Ljubljana, Ljubljana, Slovenia; ^2^ Clinical Institute of Special Laboratory Diagnostics, Children’s Hospital, University Medical Centre Ljubljana, Ljubljana, Slovenia; ^3^ Department of Laboratory Diagnostics, University Medical Centre Maribor, Maribor, Slovenia; ^4^ Department of Child, Adolescent and Developmental Neurology, Children’s Hospital, University Medical Centre Ljubljana, Ljubljana, Slovenia; ^5^ Department of Radiology, Children’s Hospital, University Medical Centre Ljubljana, Ljubljana, Slovenia; ^6^ Institute of Microbiology and Immunology, Faculty of Medicine, University of Ljubljana, Ljubljana, Slovenia; ^7^ Department of Allergology, Rheumatology and Clinical Immunology, Children’s Hospital, University Medical Centre Ljubljana, Ljubljana, Slovenia; ^8^ Department of Pediatrics, Faculty of Medicine, University of Ljubljana, Ljubljana, Slovenia

**Keywords:** Aicardi–Goutières syndrome (AGS), *IFIH1* gene, interferonopathy, Janus kinase inhibitor, combined immune deficiency

## Abstract

Aicardi–Goutières syndrome (AGS) is a genetically determined early-onset progressive encephalopathy caused by mutations leading to overexpression of type I interferon (IFN) and resulting in various clinical phenotypes. A gain-of-function (GOF) mutation in the *IFIH1* gene is associated with robust production of type I IFN and activation of the Janus kinase (JAK) signal transducer and activator of the transcription (STAT) pathway, which can cause AGS type 7. We detail the clinical case of an infant who initially presented with *Pneumocystis jirovecii* pneumonia (PCP), had recurrent respiratory infections, and was later treated with a JAK inhibitor, baricitinib, because of a genetically confirmed GOF mutation in the *IFIH1* gene. This spectrum of *IFIH1* GOF mutations with overlapping features of hyperinflammation and severe opportunistic infection, which mimics combined immunodeficiency (CID), has not been described before. In this case, therapy with baricitinib effectively blocked IFN-α activation and reduced STAT1 signaling but had no effect on the progression of the neurological disease.

## Introduction

Aicardi–Goutières syndrome (AGS) is a genetically heterogeneous disorder originally defined as an early-onset progressive encephalopathy that is characterized by intracranial calcification, white matter abnormalities, cerebral atrophy, cerebrospinal fluid (CSF) lymphocytosis, and inappropriate induction of a type I interferon (IFN)-mediated immune response, belonging to the group of type I interferonopathies (1, 2). Although AGS particularly affects the brain, immune system, and skin in the first year of life, there is a wide spectrum of disease presentations, progression, and outcomes (3). CSF and serum analyses typically exhibit increased type I IFN activity and increased levels of expression of IFN-stimulated genes in peripheral blood, the so-called IFN signature (2, 4).

As more mutations have been identified in different causative genes for AGS, it has become clear that there are significant clinical differences between patients’ phenotypes, and clinical variability has been observed even within the same genotype or family (4). In 2014, the pathogenic variant *IFIH1* (IFN induced with helicase C domain-containing protein 1 gene) was identified as the causative gene for AGS type 7 (2, 5) and accounts for only 4% of cases (6).

IFIH1 is an important intracellular sensor for various viruses; by recognizing viral double-stranded RNA, it triggers antiviral IFN responses (7). Mutations of innate immune sensor *IFIH1* are thought to be a predisposing factor for autoimmune diseases and can cause a monogenic form of systemic lupus erythematous similar to genetic overproduction of IFN- α, mutations in upstream components of the classical complement pathway, and apoptosis defects. By producing type I IFN, *IFIH1* enhances responses to its own RNA, thereby activating the adaptive immune system (7–9). The mutation in *IFIH1* may be gain of function (GOF) and can dramatically up-regulate the production of type I IFNs (5, 10, 11). Conversely, loss-of-function (LOF) variants in *IFIH1* result in a deficient antiviral defense. Therefore, it causes a primary immunodeficiency, leading to increased susceptibility to common respiratory RNA viruses (10).

The *IFIH1* GOF mutations are associated with the activation of the Janus kinase (JAK) signal transducer and activator of the transcription (STAT) pathway (5, 12). Recent evidence suggests that JAK inhibitors may be effective in blocking IFN-mediated inflammatory signaling by decreasing STAT1 phosphorylation in patients with AGS (12–15). In addition, there are some indications of possible effects on neuroinflammation and thus improvement in neurological function (12, 13). The IFN signature is an indicator of IFN signaling and might reflect disease activity (12, 13, 16). Furthermore, studies have shown that phosphorylated STAT1 (pSTAT) in T lymphocytes is greatly reduced during therapy (16).

## Case report

A male infant was born to unrelated White parents at 38 weeks of gestation by induction of labor because of oligohydramnios and intrauterine growth restriction. Birth weight was 3,050 g, birth length was 50 cm, head circumference was 34.5 cm, and Apgar scores were 9 and 9 at 1 and 5 minutes, respectively. During pregnancy, the mother had confirmed infection with the SARS-CoV-2 virus.

At 13 days of age, the patient was hospitalized for lack of weight gain and dehydration. On admission, he weighed 2,920 g, was afebrile, and had a systolic murmur below the clavicle, respiratory distress, and abnormal neurological signs. A chest radiograph showed interstitial lung infiltrates, and an echocardiogram revealed a patent foramen ovale, ductus arteriosus, and a high level of pulmonary vascular resistance. Blood test analysis revealed leukopenia, neutropenia, thrombocytopenia, and normal inflammatory parameters. He had a negative hemoculture and a nasopharyngeal swab for respiratory viruses. Screenings for congenital infections and autoimmune thrombocytopenia were also negative. Polymerase chain reaction testing for the SARS-CoV-2 virus in the placenta, umbilical blood, and CSF was negative, and the newborn also had no antibodies against SARS-CoV-2.

**Figure 1:**
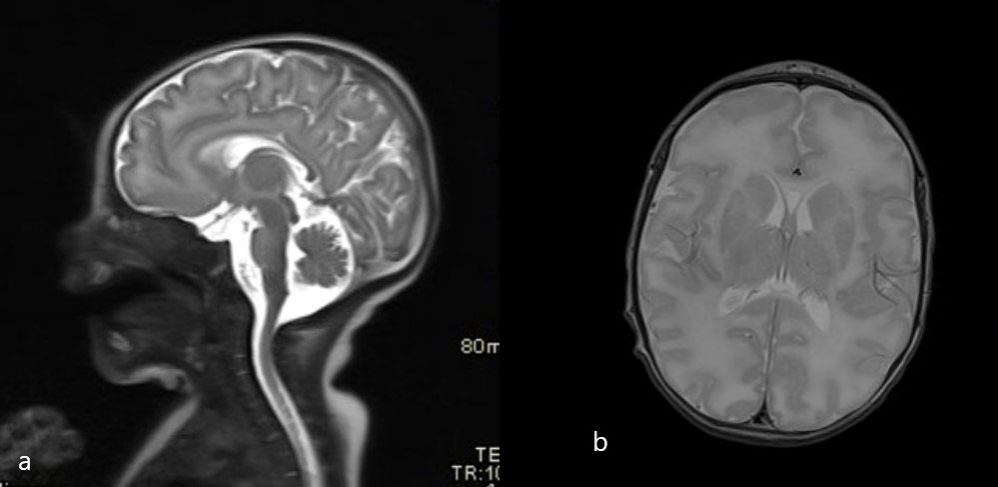
Initial brain MRI scans (T2 sagittal and axial plane). **(A)** Normal thickness of pons and midbrain. **(A, B)** Higher T2 signal of the frontal white matter.

He had abnormal neurological signs, with lethargy, hypotonia, and an abnormal spontaneous movement pattern. A cranial ultrasound showed subependymal germinolytic cysts and severe vasculopathy. Brain magnetic resonance imaging (MRI) showed diffusely slightly elevated white matter and basal ganglia signal in the T2-weighted sequences (**Figure 1**); susceptibility-weighted imaging **(**SWI) showed a hypointensive signal from slightly more prominent vessels in the basal ganglia areas.

Owing to the clinical presentation with abnormal neurological signs and neuroimaging findings, systemic inflammatory disease was suspected. Laboratory testing for interferonopathy was performed in the virology laboratory at the Hôpital Cochin, Paris, France, and showed increased IFN-α concentrations in serum (18 IU/ml) and in CSF (50 IU/ml). Whole-exome sequencing confirmed a heterozygous GOF mutation in exon 11, c.2159G>A (p.Arg720Gln), in the *IFIH1* gene (NM_022168.4). The mutation occurred *de novo* and had previously been reported in six individuals with AGS (5, 17, 18).

The patient had elevated serum immunoglobulins (Ig), with a level of IgG of 6.62 g/l, IgA of 1.32 g/l, and IgM of 1.75 g/l. He was found to have a markedly reduced concentration of B cells, T helper (Th) cells, T cytotoxic cells, and naïve CD4+ T cells. The percentages of Th1 and Th2 cells were also reduced. The percentages of activated T cells (HLA DR+) and Treg cells were markedly increased. The concentration of natural killer (NK) cells and recent thymic emigrants (RTEs) (the marker of thymic T cell production) was normal. In addition, a T-cell proliferation test (CD3/CD28 stimulated T cells) was normal.

At 2.5 months of age, the patient underwent hernioplasty of the right inguinal hernia. After surgery, he presented shortness of breath. A chest radiograph showed interstitial infiltrates on the right side. Laboratory results showed mild elevation of inflammatory parameters, lactate dehydrogenases, and significant positive β-d-glucans. A nasopharyngeal swab was positive for rhinovirus and *Pneumocystis jirovecii*. *P. jirovecii* pneumonia (PCP) was confirmed by bronchoscopy, and antibiotic treatment with trimethoprim–sulfamethoxazole was initiated. Because of recurrent mucocutaneous candidiasis, he also received systemic antifungal therapy.

**Figure 2:**
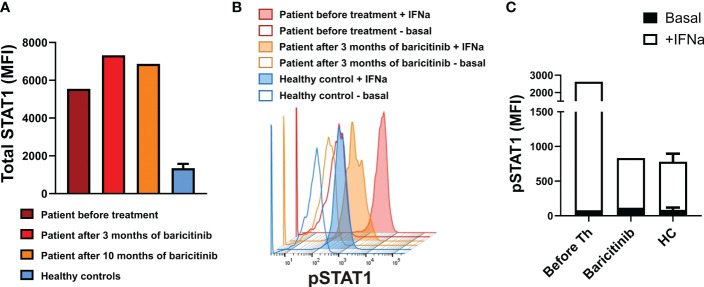
**(A)**: Total STAT1 levels in CD4+ T lymphocytes from the patient on indicated time points and healthy controls. **(B)** Phosphorylated STAT1 (pSTAT1) levels in unstimulated whole-blood CD4+ T lymphocytes and on interferon (IFN)-α induction. **(C)** Basal (unstimulated) pSTAT1 levels and pSTAT1 levels on stimulation with IFN-α for 15 minutes in CD4+ T lymphocytes from the patient before treatment and after 3 months of baricitinib, and from healthy controls. Values in **(A)** and **(C)** for healthy controls (*n*=3) are the mean ± SD. MFI, median fluorescence intensity; HC, healthy controls.

After the resolution of PCP at the age of 3.5 months, the patient was started on the JAK inhibitor baricitinib at a dosage of 0.1 mg/kg/day. Functional studies of STAT signaling (16) before initiation of baricitinib therapy revealed increased total STAT1 expression in monocytes and lymphocytes including CD4+ T cells, and increased levels of pSTAT after stimulation with IFN-α. Follow-up studies after 10 months of therapy with baricitinib revealed persistently increased total STAT1 protein levels, and there was a reduction in the level of pSTAT1 expression after stimulation with IFN-α **(** **Figure 2** **).**

At 4.5 months of age, the patient was treated for diarrhea caused by *Campylobacter jejuni* infection. At 5 months of age, he underwent an emergency right hemicolectomy owing to ischemia of the terminal ileum and ascending colon, and later underwent two relaparotomies owing to dehiscence of the ileotransverse anastomosis.

During the first year, he experienced recurrent viral respiratory infections: rhinovirus, bocavirus, coronavirus, enterovirus, and respiratory syncytial virus were detected. An increase in liver enzymes was noted during respiratory infections, which was partially attenuated by continuing baricitinib therapy during the infections.

After 1 year most immune parameters reached normal levels: concentrations of B cells, T helper cells, T cytotoxic cells, and naive CD4+ T cells and percentage of Th1 and Th2 cells. Only the percentage of Treg cells remained increased.

**Figure 3:**
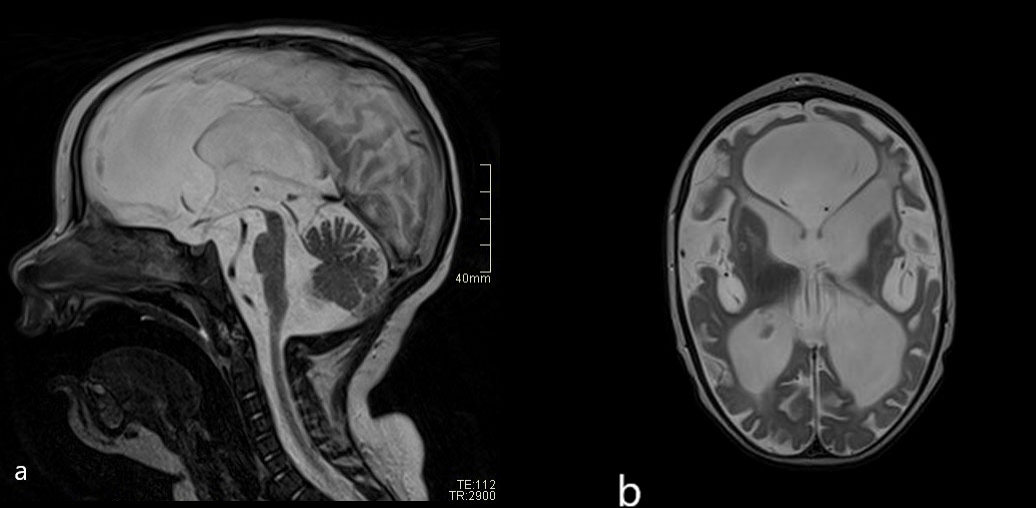
**(A)** Follow-up brain MRI scans (T2 sagittal and axial plane) after 1 year: severe atrophy of midbrain compared with initial MRI. **(B)** Severe reduction of brain volume and abnormal signal of the white matter—leucoencephalopathy.

The neuroimaging results and abnormal neurological signs pointed us to an early diagnosis and initiation of treatment. At a neurological assessment at 3 months of age, the child presented with axial hypotonia and increased muscle tone of the extremities. Treatment with tiagabine and B vitamins was initiated, together with baricitinib. A neurological evaluation at 6 months of age showed decreased growth in head circumference, poor eye contact, and limb spasticity, with occasional dystonia. The patient was unable to hold up his head, and spontaneous movements were poorly expressed, without meaningful coordination. Further neurological follow-ups showed the developmental delay to be severe. A brain MRI scan at 12 months of age showed marked atrophy of the deep and subcortical white matter of both hemispheres, with enlarged ventricles, sulci, and subarachnoid spaces. The brainstem and cerebellar peduncles were also greatly reduced in volume. In the reduced basal nuclei, signs of calcifications were reported **(** **Figure 3** **).** At the age of 1.5 years, global developmental delay was identified, with intellectual disability and without any signs of speech development. Epileptic seizures with twitching in the left arm and short absences had occurred. An electroencephalogram (EEG) showed multifocal epileptiform discharges. Levetiracetam was introduced. Feeding problems became more frequent and, consequently, a percutaneous gastrostoma was introduced. The child and his family were also receiving follow-up treatment from the pediatric palliative care team.

## Discussion

We have detailed the case of an infant with early-diagnosed AGS type 7 with genetically confirmed heterozygous GOF mutation in the *IFIH1* gene who initially presented neurological manifestations as well as *P. jirovecii* pneumonia (PCP) resembling combined immunodeficiency (CID). The patient was later treated with a Janus kinase (JAK) inhibitor, baricitinib, which reduced STAT1 signaling but had no effect on the progression of the neurological disease.

The initial clinical presentation of our patient was suggestive of prenatal onset of the disease and raised suspicion of interferonopathies. In a large case series of AGS patients, only 11.4% of infants had abnormal neurological signs at birth without obvious systemic signs, and others developed symptoms later in life, usually within the first year. Notably, individuals with an *IFIH1* mutation had normal development in the first year of life and often did not present until after 1 year (4). Rice et al. reported that clinical presentation at or shortly after birth is associated with a severe AGS phenotype, suggesting prenatal onset of the disease (17).

A heterozygous c.2159G>A (p.Arg720Gln) mutation in the *IFIH1* gene (NM_022168.4) has been previously identified in six individuals with a severe AGS phenotype and its functional effect has been demonstrated by IFN scoring (2, 5, 17, 18). Rice et al. reported two patients with intrauterine growth restriction and thrombocytopenia at birth who had severe developmental delay. MRI at 8 and 12 months of age showed cerebral atrophy with calcifications in the basal ganglia and white matter disease. The first patient developed seizures at 13 months of age and died of pneumonia at 2 years of age. The second patient had hypertrophic cardiomyopathy and nephrotic syndrome diagnosed at 7 and 10 months of age, respectively (5). Subsequently, Adang et al. described a male patient with the same mutation, who also presented in the neonatal period with hepatosplenomegaly and severe thrombocytopenia, had profound developmental delay, and developed severe pulmonary hypertension by the age of 2 years (18). Moreover, other studies have reported various amino acid substitutions in *IFIH1* mutation-positive patients (3, 11, 18–22). Recent studies suggest that IFN status assessment is a reliable disease marker (13, 17), and a positive correlation has been found between IFN activity in CSF measured within 1 year of disease onset and the degree of subsequent disability (4). In this case, analysis of CSF and serum showed increased IFN type I and an enhanced IFN signature.

*P. jirovecii* is an opportunistic microbial pathogen that is particularly threatening in immunocompromised individuals and can cause PCP (23). To our knowledge, PCP with a mutation in *IFIH1* has not previously been reported. Our patient also had persistent mucocutaneous candidiasis and numerous recurrent respiratory viral infections as clinical signs of immunodeficiency. The deficient antiviral defense has been described only in *IFIH1* LOF mutations (10), but even LOF mutations are not associated with opportunistic infections. CID-like presentation with PCP and severe T-cell lymphopenia was previously reported in two patients with stimulator of interferon genes (STING)-associated vasculopathy with onset in infancy (SAVI), which is also a type I interferonopathy and is caused by a GOF mutation in the transmembrane protein 173 gene (*TMEM173*) encoding STING (24, 25). Based on the disease course in our case, it appears that patients with AGS with the *IFIH1* GOF mutation may present similarly to patients with CID, with opportunistic infections associated with autoimmune and hyperinflammatory manifestations.

Previous studies have shown that JAK inhibitors can effectively block the activation of IFNs in patients with AGS (13), especially in patients with *IFIH1*-related disease (12). IFN signature and STAT1 phosphorylation in T lymphocytes could serve as indicators of response to treatment (12, 13, 16). In our patient, we started treatment with baricitinib, a JAK1 and JAK2 inhibitor, in the first months after birth, using the recommended dosage (13). During treatment, we noted a significant functional improvement, a significant reduction in the IFN signature, and a reduction in pSTAT in peripheral blood T lymphocytes. Studies of STAT signaling in our patient were performed as previously reported (16), including analysis of total STAT1 expression in T cells at different time points without technical repeats.

Some recent studies have reported improvement of neurological functions after JAK inhibitor therapy, even in patients with severe and prolonged disease (13, 26), suggesting that treatment in the earliest stages of the disease could lead to important clinical gains (27). By contrast, our patient experienced severe progression of encephalopathy in spite of early diagnosis and treatment with baricitinib, which was confirmed by neuroimaging at 12 months (28, 29). Our findings indicate that JAK inhibitors effectively blocked the systemic IFN-mediated inflammatory response but had little or no effect on the brain disease. Based on the functional studies of STAT signaling, our data suggest that the measurement of STAT1 phosphorylation can be used as a useful monitoring tool for adjusting the dosage of baricitinib. No common adverse effects were reported (16), and treatment was well tolerated by our patient.

## Conclusion

Our case highlights the phenotypic heterogeneity of AGS. PCP and recurrent respiratory infections have not previously been described as part of the clinical spectrum associated with a GOF mutation in the *IFIH1* gene. This case shows that AGS with GOF mutation in the *IFIH1* gene could mimic CID with opportunistic infections associated with autoimmune and hyperinflammatory manifestations. In our experience, baricitinib effectively blocks IFN-α activation, but even early treatment with a JAK inhibitor failed to halt the progression of neurological manifestations.

## Data availability statement

The raw data supporting the conclusions of this article will be made available by the authors, without undue reservation.

## Ethics statement

Ethics review and approval were not required for the study on human participants in accordance with the local legislation and institutional requirements. Written informed consent to participate in this study was provided by the participants’ legal guardian/next of kin. Written informed consent was obtained from the minor(s)’ legal guardian/next of kin for the publication of any potentially identifiable images or data included in this article.

## Author contributions

MŽ, ASŠ, NŠ, and TA contributed to patient care and treatment. MŽ, ASŠ, and TA wrote the manuscript and reviewed the literature. DK contributed to interpreting and describing the imaging findings. MD performed genetic evaluation. AG and AI contributed to immunological evaluation and functional testing. All authors contributed to diagnostic procedure, manuscript revision, and read, and approved the submitted version.
